# Effects of a home-visit nursing simulation program for frailty prevention in older adults on nursing competencies: a quasi-experimental pre–post study

**DOI:** 10.3389/fmed.2026.1785752

**Published:** 2026-06-17

**Authors:** HyoEun Park, GyeongSook Park, HwaYoung Kim

**Affiliations:** 1 Department of Nursing, School of Health Science, Hanseo University, Seosan, Republic of Korea; 2 Department of Nursing, Kunjang University College, Gunsan, Republic of Korea; 3 Department of Nursing, Mokpo Catholic University, Mokpo, Republic of Korea

**Keywords:** clinical judgment, clinical performance, frailty prevention, self-efficacy, simulation nursing education

## Abstract

**Introduction:**

Developing effective educational strategies to enhance nursing competencies is essential, particularly in community health settings. This study developed a home-visit nursing simulation program for frailty prevention in older adults and evaluated its effectiveness among nursing students.

**Methods:**

A quasi-experimental one-group pre–post design was used. Participants were 50 undergraduate nursing students. Repeated measures ANOVA, Pearson’s correlation analysis, and multiple regression analysis were conducted.

**Results:**

Clinical judgment and clinical performance showed significant improvements after the simulation, whereas self-efficacy did not show a statistically significant change. Simulation quality was positively associated with clinical judgment and clinical performance.

**Discussion:**

These findings suggest that home-visit simulation may be a useful educational strategy for enhancing clinical judgment and clinical performance in nursing education. Self-efficacy appeared to be more closely associated with major satisfaction than with simulation quality.

## 1 Introduction

With the advent of an aging society, frailty has emerged as one of the most pressing health issues among older adults. Frailty is a multidimensional concept involving declines in physical, psychological, and social functioning, which can lead to adverse outcomes such as falls, hospitalization, and mortality (1, 2). In Korea, where the proportion of older adults is rapidly increasing, strengthening professional nursing competencies to effectively prevent and manage frailty is essential. Nursing students, who will serve as the future frontline workforce directly caring for frail older adults, must therefore be equipped with appropriate competencies, making this an important educational priority.

The prevalence of frailty, including pre-frailty, among adults aged 65 years and older is reported to be approximately 39–42% both domestically and internationally (3, 4). Furthermore, frail older adults are six times more likely to die within 3 years compared to their non-frail counterparts (5, 6). Thus, identifying determinants of frailty and implementing preventive interventions are critical nursing tasks to reduce mortality risk.

Given the complex and multifactorial health needs of older adults, effective community-based care requires nurses to possess well-developed competencies that extend beyond disease-focused treatment to the maintenance and improvement of functional ability, particularly in the context of frailty (7). Accordingly, strengthening home-visiting nursing competencies is essential, and providing such training during undergraduate nursing education is especially important. However, in actual community health nursing practicums, opportunities for home-visit training have been increasingly limited due to growing student enrollment, community resistance, concerns regarding privacy and safety, and heightened risks of infectious diseases such as COVID-19. As a result, many practicums rely heavily on observational and passive learning experiences rather than active skill development (8–10).

Simulation-based education has been highlighted as an alternative to address these challenges. By recreating realistic clinical situations and incorporating structured debriefing, simulation enables students to integrate knowledge, skills, and attitudes, thereby improving competencies such as critical thinking, clinical judgment, self-efficacy, clinical performance, and satisfaction (11–13). Nevertheless, most simulation studies in nursing education have focused on fundamental, emergency, maternal, and pediatric nursing, while community health nursing simulations, particularly those addressing frailty, remain scarce. Indeed, a survey reported that only 4.8% of simulation modules addressed community health nursing, underscoring the quantitative and qualitative insufficiency of such training (14). Despite the growing recognition of simulation as an innovative pedagogical strategy, its theoretical foundations and practical applications in nursing education are not always sufficiently articulated. Previous studies have demonstrated the effectiveness of simulation-based education across various nursing courses and among nursing students; however, the extent to which these approaches have been systematically applied in community health nursing remains limited. Therefore, a more detailed examination of simulation as an educational strategy, supported by recent empirical evidence, is warranted (15–17).

Therefore, this study aimed to develop a home-visit nursing simulation program focused on frailty prevention among older adults and to evaluate its effectiveness using Jeffries’ Simulation Model in terms of clinical judgment, self-efficacy, and clinical performance. This study is expected to provide empirical evidence that may support the development of nursing students’ competencies in caring for frail older adults. In doing so, it seeks to contribute to the improvement of community health nursing education and to inform the preparation of professional nurses in the context of an aging society.

### 1.1 Purpose of the study

The purpose of this study was to evaluate the effects of a frailty-prevention simulation program on nursing students’ competencies, specifically clinical judgment, self-efficacy, and clinical performance. The specific objectives were as follows:

1. To assess the perceived quality of simulation-based education after the program.
2. To compare differences in nursing student competencies before and after simulation practice.
3. To examine the correlations between the quality of simulation-based education and nursing competencies.
4. To determine the effects of the quality of simulation-based education on nursing competencies.

## 2 Materials and methods

### 2.1 Research design

This study employed a one-group pretest–posttest design to examine the effects of simulation education using a standardized patient on nursing students’ competencies (clinical judgment, self-efficacy, and clinical performance).

### 2.2 Theoretical framework

This study was guided by the Jeffries Simulation Model (11, 18), which consists of four core components: learners, the quality of simulation-based education (educational design, educational operation, and educational practice), nursing student competencies (clinical judgment, self-efficacy, and clinical performance in home-visit nursing), and educational satisfaction.

First, the learners in this study were fourth-year undergraduate nursing students who were expected to actively engage in the simulation program and perform their assigned nursing roles in a self-directed manner.

Second, the quality of simulation-based education was evaluated across three subdomains:

- Educational design (QoSED): Refers to the structural aspects of effective simulation scenarios, including objectives, fidelity, problem-solving, cues, and debriefing (18). In this study, clear learning objectives were provided for home-visit nursing in frailty prevention, standardized patients were used to enhance fidelity, multiple cues such as pre-assessment data were offered to facilitate clinical reasoning, and structured debriefing promoted reflective thinking.
- Educational operation (QoSEO): Represents teaching efficiency, in which instructors facilitate learning by creating supportive environments and managing the simulation effectively. This includes class organization, teaching methods, instructional materials, and fairness of evaluation (19, 20). In this study, lessons were pre-announced, audiovisual materials were provided, evaluation criteria were communicated in advance, and constructive feedback was offered.
- Educational practice (QoSEP): Involves active learning, feedback, learner–faculty interaction, and collaboration (11, 18). Unlike traditional lecture-centered teaching, simulation places learners at the center while faculty serve as facilitators and evaluators, supporting student learning and providing feedback (21).

Third, nursing competencies represent the expected outcomes of simulation education, encompassing clinical judgment, self-efficacy, and clinical performance. Previous studies have demonstrated that simulation enhances clinical judgment (22, 23), improves self-efficacy through experiential learning (24, 25), and strengthens clinical performance (18, 26).

Theoretical framework of the study based on the Jeffries Simulation Model. The framework illustrates the relationships among learners (4th-year nursing students), the quality of simulation-based education (educational design, educational operation, and educational practice), and the expected outcomes. The quality of simulation-based education influences both nursing competencies (clinical judgment, self-efficacy, and clinical performance) and educational satisfaction.

### 2.3 Research participants

Participants were 50 undergraduate nursing students enrolled in a university located in Gyeonggi Province, South Korea. Eligible participants were those who had completed the theoretical course in adult nursing. Recruitment was conducted through convenience sampling between July 7 and July 10, 2025. The researcher visited the university, explained the study purpose and procedures to faculty members, and obtained cooperation. Recruitment announcements were then posted on the university bulletin board and social media. Students who met the inclusion criteria were invited for face-to-face consultations, during which the study purpose, procedures, and randomization process were explained. Written informed consent was obtained prior to enrollment.

The required sample size was calculated using G*Power 3.1.9.7 (27). Based on a previous study (28), with an effect size of 0.80, a significance level of 0.04, and a power of 0.80 for a two-tailed independent *t*-test, a minimum of 50 participants was required. Considering a 20% dropout rate (29), a total of 56 participants were recruited. Ultimately, six students withdrew during the program, leaving 50 participants included in the final analysis.

### 2.4 Scenario development and simulation implementation

For this study, a simulation scenario for frailty prevention in community-dwelling older adults was developed to guide instructors, students, and standardized patients. The scenario was titled “Home Visiting Nursing for Pre-frail Older Adults in the Community” and focused on therapeutic communication, nursing assessment, and nursing interventions. The scenario was developed in accordance with established simulation education principles and was informed by the INACSL Standards of Best Practice: Simulation^SM^ to ensure educational quality and consistency.

The scenario was structured in three stages, incorporating essential nursing practices such as frailty assessment, nutritional assessment and dietary education, physical activity assessment and exercise guidance, supportive nursing to prevent social isolation, utilization of community resources, and reinforcement of community participation. Each stage was designed to help nursing students identify potential risk factors in pre-frail older adults and implement preventive interventions.

The components of the scenario included a module overview, pre-briefing, scenario flowchart, debriefing, and evaluation tools. The module overview provided the simulation context, patient information, learning objectives, missions, pre-questions, learning outcomes, prerequisite knowledge, and related fundamental nursing skills. The pre-briefing introduced the initial home visit situation and allowed students to classify nursing problems by priority and plan corresponding interventions. The scenario flowchart described the sequence of interactions between the student and the standardized patient, including prompts and guiding questions. Finally, the debriefing was structured according to Steinwachs’ (30) three phases—description, analysis, and application—enabling students to reflect on their performance and reinforce their learning outcomes.

The simulation intervention, including scenario implementation and debriefing, was conducted directly by the principal investigator. Debriefing consisted of both individual and group debriefing sessions, conducted using a structured and standardized format to ensure consistency across participants.

Each simulation session was structured as a 60-min program, comprising 20 min of pre-learning, 20 min of simulation, and 20 min of debriefing. The simulation scenario was designed for one student at a time, reflecting the real-world context of community health nursing practice in Korea, where public health nurses typically conduct home visits independently. Accordingly, the simulation environment was developed to closely replicate a home-visiting nursing setting.

Student performance was evaluated by the principal investigator and a trained standardized patient using predefined assessment criteria. Prior to the study, the standardized patient underwent structured training based on the scenario script and evaluation guidelines to ensure consistency in role portrayal and scoring across sessions. To further enhance reliability, standardized procedures were applied throughout the evaluation process.

Outcome measures were assessed using a pre-test administered immediately before the simulation and a post-test conducted immediately after the simulation to capture changes associated with participation.

### 2.5 Standardized patient (SP) training and video preparation

To enhance the effectiveness of the simulation education, a standardized patient (SP) scenario and guideline were developed. In accordance with the simulation scenario, background scenes of community-dwelling pre-frail older adults and home-visit settings were pre-recorded. One SP was recruited for the program, a 63-year-old woman with more than 10 years of experience as a standardized patient in medical and nursing education, and with a strong understanding of nursing student practice.

Prior to the simulation, the SP reviewed the script and participated in three preparatory sessions to ensure familiarity with the role and to refine natural interactions. The script was revised based on these rehearsals to improve realism. Additional practice was then conducted using the revised script, followed by the production of 15-min demonstration videos. During the filming, the principal investigator played the role of a visiting nurse to ensure that the evaluation criteria for the simulation were clearly demonstrated and aligned with the study objectives.

### 2.6 Research variables

#### 2.6.1 General characteristics

In this study, the general characteristics of nursing students included gender, age, satisfaction with the nursing major, and prior experience with simulation-based education. All participants were female (100%). The mean age of participants was 21.84 ± 0.78 years (range: 21–23).

#### 2.6.2 Quality of simulation-based education (QoSE)

##### 2.6.2.1 Quality of simulation-based educational design (QoSED)

The quality of simulation-based educational design was measured using the Simulation Design Scale (SDS) originally developed by the National League for Nursing (NLN) and adapted into Korean by Yoo (31) with permission. The tool consists of 21 items across five domains: learning objectives and content (6 items), support (4 items), problem solving (5 items), feedback (4 items), and fidelity (2 items). Each item is rated on a 5-point Likert scale, with higher scores indicating better quality of simulation design. The reliability of the original tool was Cronbach’s α = 0.92, Yoo’s version α = 0.90, and in this study Cronbach’s α = 0.96.

##### 2.6.2.2 Quality of simulation-based education operation (QoSEO)

The quality of simulation-based operation was assessed using a teaching efficiency tool originally developed by Reeve (19), revised by Choi (32), and further modified for simulation-based education by Park and Ji (20). The tool consists of 10 items in three domains: class management (2 items), teaching methods and materials (5 items), and objectivity of class evaluation (3 items). Items are rated on a 5-point Likert scale, with higher scores indicating higher teaching efficiency. Cronbach’s α was 0.93 in Reeve (19), 0.96 in Park and Ji (20), and 0.91 in this study.

##### 2.6.2.3 Quality of simulation-based educational practice (QoSEP)

The quality of simulation-based practice was measured using the Educational Practices Questionnaire (EPQ) developed by the NLN and translated/adapted into Korean by Baek (33). The instrument consists of 16 items in four domains: active learning, collaboration, diverse ways of learning, and high expectations. Each item is rated on a 5-point Likert scale, with higher scores reflecting better quality of educational practices. Cronbach’s α was 0.86 in the original study, 0.91 in Baek (33), and 0.95 in this study.

#### 2.6.3 Nursing competencies

##### 2.6.3.1 Clinical judgment

Clinical judgment was measured using the Lasater Clinical Judgment Rubric (LCJR) developed by Lasater (32) and translated into Korean by Sim (34), with permission. This tool uses a 4-point Likert scale, where higher scores indicate greater clinical judgment ability. Cronbach’s α was 0.88 in the original tool and 0.89 in this study.

##### 2.6.3.2 General self-efficacy

General self-efficacy was measured using the Self-Efficacy Scale (SES) developed by Sherer et al. (35) and revised by Hong (36). Among the 23 items, 17 items assessing general self-efficacy were used in this study. Each item is rated on a 5-point Likert scale, with higher scores reflecting greater self-efficacy. Thirteen negatively worded items were reverse-coded. Cronbach’s α was 0.86 in Hong (36) and 0.89 in this study.

##### 2.6.3.3 Clinical performance

Clinical performance was measured using the Six-Dimension Scale of Nursing Performance originally developed by Curran et al. (37) and revised by Choi (32). The tool consists of 20 items across five domains, rated on a 5-point Likert scale. Higher scores indicate greater nursing performance. Content validity was confirmed by six experts (two community health nursing professors, one gerontological nursing professor, two public health center visiting nurses, and one frailty-focused doctoral-level nurse researcher). The content validity index (CVI) for all items was 1.00. Cronbach’s α was 0.96 in the original study and 0.96 in this study.

#### 2.6.4 Practice satisfaction

Practice satisfaction was measured using a tool originally developed by Yoo (31) to assess learning satisfaction, modified by replacing the term “learning” with “practice” and excluding items related to instructor satisfaction. The tool is rated on a 5-point Likert scale, with higher scores indicating greater practice satisfaction. Cronbach’s α was 0.86 in Yoo (31) and 0.91 in this study.

### 2.7 Ethical considerations

This study was approved by the Institutional Review Board (IRB) of Hanseo University, which is affiliated with the researchers (IRB No. HS-2025-39). All methods were performed in accordance with the relevant guidelines and regulations. Individuals who voluntarily agreed to participate in the study were provided with a clear explanation regarding the objectives, procedures, and voluntary nature of participation. Written informed consent was obtained electronically from all participants prior to their inclusion in the study.

### 2.8 Statistical analysis

The data were analyzed using the following procedures:

First, frequency analysis was conducted to examine the general characteristics of the participants.

Second, descriptive statistics were used to identify the levels of perceived quality of simulation-based education, nursing competencies, and practice satisfaction among nursing students.

Third, repeated measures analysis of variance (ANOVA) was performed to verify whether nursing competencies significantly changed after participating in simulation education compared with before.

Fourth, Pearson’s correlation analysis was used to examine the relationships between the quality of simulation-based education and nursing competencies.

Finally, multiple regression analysis was conducted to test the effects of perceived simulation education quality on nursing competencies.

A *post hoc* power analysis was conducted, indicating that the sample size was sufficient to detect meaningful effects.

The assumptions of normality and homogeneity of variance for ANOVA were assessed and met.

Prior to conducting the regression analysis, key assumptions were assessed. Linearity between independent and dependent variables was examined, and independence of errors was confirmed. The normality of residuals and homoscedasticity were evaluated and met. Multicollinearity was assessed using variance inflation factor (VIF) and tolerance values, and no serious multicollinearity was identified. Confidence intervals were considered in the interpretation of the results.

All statistical analyses were performed using SPSS Statistics version 26.0 (IBM Corp., Armonk, NY, United States), and a significance level of *p* < 0.05 was applied.

## 3 Results

### 3.1 General characteristics of participants

Frequency analysis was conducted to examine the general characteristics of the participants. The results showed that 20 students (40.0%) were 21 years old, 18 students (36.0%) were 22 years old, and 12 students (24.0%) were 23 years or older. Regarding satisfaction with their nursing major, no student reported being “very dissatisfied” (0%), while 3 students (6.0%) reported “dissatisfied,” 11 students (22.0%) “neutral,” 26 students (52.0%) “satisfied,” and 10 students (20.0%) “very satisfied,” indicating that more students were satisfied than dissatisfied with their major. In addition, 31 students (62.0%) had previous experience with simulation-based education. The general characteristics of participants are presented in Table 1.

**TABLE 1:** General characteristics of participants.

Characteristics	Categories	*n*	%
Age (years)			
	21	20	40.0
	22	18	36.0
	≥ 23	12	24.0
Satisfaction with major			
	Very dissatisfied	0	0.0
	Dissatisfied	3	6.0
	Neutral	11	22.0
	Satisfied	26	52.0
	Very satisfied	10	20.0
Previous simulation experience			
	Yes	31	62.0
	No	19	38.0
Total	50	100.0

### 3.2 Quality of Simulation-based education, nursing competencies, and practice satisfaction

Descriptive statistics were conducted to assess nursing students’ perceptions of the quality of simulation-based education, nursing competencies, and practice satisfaction. All variables were measured on a 5-point Likert scale. The mean score for the overall quality of simulation-based education was 4.46, with subdomains as follows: educational design (*M* = 4.40), educational operation (*M* = 4.56), and educational practice (*M* = 4.48), indicating generally high levels. Among the nursing competencies, clinical judgment had a mean of 4.10, self-efficacy 3.67, and clinical performance 4.42. Practice satisfaction was also high, with a mean of 4.45. The descriptive statistics are presented in Table 2.

**TABLE 2:** Quality of simulation-based education, nursing competencies, and practice satisfaction.

Variables	Possible range	Mean	SD	Skewness	Kurtosis
Quality of simulation-based education	1–5	4.46	0.44	−0.43	−0.77
Educational design	1–5	4.40	0.49	−0.60	0.41
Educational operation	1–5	4.56	0.45	−0.42	−1.46
Educational practice	1–5	4.48	0.50	−0.74	−0.17
Clinical judgment	1–5	4.10	0.38	−0.08	0.07
Self-efficacy	1–5	3.67	0.63	−0.04	−0.71
Clinical performance	1–5	4.42	0.52	−0.62	−0.07
Practice satisfaction	1–5	4.45	0.60	−0.72	−0.64

To verify the assumption of normality, skewness and kurtosis were examined. The results showed that skewness values were all within ± 2 and kurtosis values within ± 7, suggesting that the data satisfied the assumption of normal distribution (38) and were appropriate for subsequent statistical analyses.

### 3.3 Changes in nursing competencies before and after simulation education

Repeated measures ANOVA was conducted to examine whether nursing competencies significantly changed before and after participation in simulation education. The results were as follows:

The mean score for clinical judgment increased from 3.86 at pre-test to 4.10 at post-test, showing a mean difference of 0.24, which was statistically significant (*F* = 23.25, *p* < 0.001), indicating a large effect size. Self-efficacy increased from 3.59 at pre-test to 3.67 at post-test, a difference of 0.08, which was not statistically significant. Clinical performance increased from 4.12 at pre-test to 4.42 at post-test, showing a mean difference of 0.31, which was statistically significant (*F* = 21.04, *p* < 0.001).

In summary, both clinical judgment and clinical performance significantly improved after simulation education, whereas self-efficacy did not show a statistically significant change. Changes in nursing competencies before and after simulation education are shown in Table 3.

**TABLE 3:** Changes in nursing competencies before and after simulation education.

Variables	Pre-test	Post-test	Change score	*F*	*p*
Clinical judgment	3.86 ± 0.33	4.10 ± 0.38	0.24 ± 0.35	23.25	< 0.001
Self-efficacy	3.59 ± 0.48	3.67 ± 0.63	0.08 ± 0.52	1.33	0.254
Clinical performance	4.12 ± 0.53	4.42 ± 0.52	0.31 ± 0.47	21.04	< 0.001

### 3.4 Correlations between the quality of simulation-based education and nursing competencies

Pearson’s correlation analysis was conducted to examine the relationships between the quality of simulation-based education and nursing competencies among nursing students.

The quality of simulation-based education showed a significant positive correlation with clinical judgment (*r* = 0.43, *p* = 0.002). Subdomains of educational design (*r* = 0.42, *p* = 0.002), educational operation (*r* = 0.35, *p* = 0.013), and educational practice (*r* = 0.38, *p* = 0.006) were also significantly correlated with clinical judgment.

No significant correlations were found between the quality of simulation-based education and self-efficacy, and none of the subdomains (educational design, operation, and practice) were significantly related to self-efficacy.

The quality of simulation-based education demonstrated a significant positive correlation with clinical performance (*r* = 0.65, *p* <0.001). Similarly, educational design (*r* = 0.59, *p* < 0.001), educational operation (*r* = 0.52, *p* < 0.001), and educational practice (*r* = 0.62, *p* < 0.001) were all significantly correlated with clinical performance. Correlations among the variables are presented in Table 4.

**TABLE 4:** Correlations between the quality of simulation-based education and nursing competencies.

Quality of simulation-based education	Educational design	Educational operation	Educational practice	Clinical judgment	Self-efficacy	Clinical performance	
r (p)	r (p)	r (p)	r (p)	r (p)	r (p)	r (p)	
1. Quality of simulation-based education	1	1	1	1	1	1	1
1.1 Educational design	0.94 (< 0.001)						
1.2 Educational operation	0.75 (< 0.001)	0.54 (< 0.001)					
1.3 Educational practice	0.96 (< 0.001)	0.85 (< 0.001)	0.68 (< 0.001)				
2. Clinical judgment	0.43 (0.002)	0.42 (0.002)	0.35 (0.013)	0.38 (0.006)			
3. Self-efficacy	0.16 (0.274)	0.11 (0.446)	0.12 (0.401)	0.20 (0.165)	0.37 (0.009)		
4. Clinical performance	0.65 (< 0.001)	0.59 (< 0.001)	0.52 (< 0.001)	0.62 (< 0.001)	0.46 (0.001)	0.21 (0.145)	

### 3.5 Effects of the quality of simulation-based education on nursing competencies

Multiple regression analysis was conducted to examine the effects of the quality of simulation-based education on nursing competencies, while controlling for age, satisfaction with the nursing major, and prior experience with simulation-based education. Due to high intercorrelations among the subdomains of educational design, operation, and practice, the overall quality score was entered as the independent variable to avoid multicollinearity.

The regression model for clinical judgment explained approximately 24% of the variance, and the quality of simulation-based education had a significant positive effect on clinical judgment (β = 0.36, *p* = 0.013). This indicates that higher perceived quality of simulation-based education was associated with higher levels of clinical judgment.

The regression model for self-efficacy explained approximately 23% of the variance. Among the control variables, satisfaction with the nursing major showed a significant positive effect on self-efficacy (β = 0.41, *p* = 0.005), whereas the quality of simulation-based education itself was not a significant predictor. This suggests that self-efficacy was more strongly associated with major satisfaction than with the quality of simulation-based education.

The regression model for clinical performance explained approximately 50% of the variance, indicating the highest explanatory power among the competencies. Both satisfaction with the nursing major (β = 0.27, *p* = 0.021) and the quality of simulation-based education (β = 0.58, *p* < 0.001) had significant positive effects on clinical performance. Thus, students with higher major satisfaction and higher perceptions of simulation education quality demonstrated stronger clinical performance.

Due to high intercorrelations among the subdomains of educational design, operation, and practice, the overall quality score was entered as the independent variable to avoid multicollinearity. Multicollinearity was assessed using variance inflation factor (VIF) and tolerance values, and no serious multicollinearity was identified. The results of the multiple regression analyses are presented in Table 5.

**TABLE 5:** Effects of the quality of simulation-based education on nursing competencies.

Dependent variable	Independent variables	*B*	SE	β	*t*	*p*	*R*^2^ (Adj *R*^2^)	*F* (*p*)
Clinical judgment	Age	−0.01	0.02	−0.04	−0.29	0.770	0.24 (0.17)	3.51 (0.014)
	Satisfaction with major	0.11	0.07	0.24	1.73	0.091		
	Prior simulation experience	0.00	0.10	0.00	0.01	0.991		
	Quality of simulation-based education	0.31	0.12	0.36	2.60	**0.013**		
Self-efficacy	Age	0.04	0.03	0.17	1.29	0.205	0.23 (0.16)	3.38 (0.017)
	Satisfaction with major	0.32	0.11	0.41	2.95	**0.005**		
	Prior simulation experience	−0.04	0.17	−0.03	−0.23	0.816		
	Quality of simulation-based education	0.07	0.20	0.05	0.34	0.732		
Clinical performance	Age	0.00	0.02	−.01	−0.06	0.949	0.50 (0.45)	11.16 (< 0.001)
	Satisfaction with major	0.17	0.07	0.27	2.40	**0.021**		
	Prior simulation experience	0.09	0.11	0.09	0.82	0.415		
	Quality of simulation-based education	0.69	0.13	0.58	5.17	**< 0.001**		

Bold values indicate statistically significant results (*p* < 0.05). B, unstandardized coefficient; SE, standard error; β, standardized coefficient.

## 4 Discussion

### 4.1 Effectiveness of simulation-based education for frailty prevention

This study examined changes in clinical judgment, self-efficacy, and home-visit clinical performance following participation in a simulation-based education program for frailty prevention. The findings indicated that all three outcomes showed significant improvements within the intervention group, suggesting the potential educational value of simulation for frailty prevention in community and home-visit nursing contexts.

First, clinical judgment improved significantly after the intervention. This finding is consistent with previous studies reporting no significant superiority of simulation over traditional lecture-based education in terms of clinical judgment outcomes (20, 39, 40). Nevertheless, other studies have shown that simulation-based education can enhance clinical judgment by providing structured learning experiences that promote reasoning and reflection (22, 23, 41, 42). In the present study, the use of structured guidelines and repeated reflective debriefing likely contributed to improvements in clinical judgment over time. However, because the home-visit simulation emphasized communication and interaction rather than urgent medical decision-making, the magnitude of change in clinical judgment may have been limited. Future studies should therefore consider incorporating more complex clinical scenarios and advanced debriefing strategies to further strengthen clinical judgment development.

Second, self-efficacy also showed a significant improvement following participation in the simulation-based education program. This result is consistent with prior research suggesting that simulation may enhance nursing students’ confidence by providing safe and structured opportunities for practice (24, 43, 44). The educational design of this study—including pre-briefing, interactions with standardized patients, and reflective journaling—may have facilitated positive self-evaluation, learning motivation, and confidence. In particular, authentic patient interactions may have allowed students to recognize and address patient problems in a controlled environment, thereby supporting the development of self-efficacy (45, 46). These findings further support existing evidence that simulation-based education may offer safe and repetitive practice opportunities that can foster confidence among nursing students (47, 48).

Third, home-visit clinical performance improved significantly after the intervention, indicating that the simulation-based education program may have contributed to improvements in students’ practical nursing skills. This finding aligns with previous studies and meta-analyses reporting improvements in clinical performance following simulation training (18, 26, 49–51). In this study, the systematic design of simulation scenarios based on the nursing process, the realism provided by standardized patients, and structured debriefing sessions likely contributed to improvements in clinical performance. Moreover, the observed positive correlation between self-efficacy and clinical performance (22, 52) suggests that increases in self-efficacy may play a role in facilitating improvements in clinical performance over time.

### 4.2 Influence of perceived quality of simulation-based education on nursing competencies

This study also examined the impact of perceived simulation quality on nursing competencies. Multiple regression analysis revealed that simulation quality significantly influenced clinical judgment and clinical performance, but not self-efficacy. In contrast, satisfaction with the nursing major was found to positively affect both self-efficacy and clinical performance.

First, the finding that simulation quality positively affected clinical judgment is consistent with prior studies, suggesting that higher quality in educational design, delivery, and debriefing enhances students’ ability to recognize and reason through patient problems (41, 42, 53). This reflects the ability of simulation to reproduce clinical situations, offering students opportunities to practice reasoning and engage in reflection within a safe environment.

Second, the absence of a significant effect of simulation quality on self-efficacy, although unexpected, may be explained by the fact that self-efficacy depends more strongly on personal motivation and major satisfaction than on the educational process itself (24, 44). In this study, major satisfaction significantly predicted self-efficacy, indicating that students’ sense of belonging and pride in their nursing major influenced their confidence and self-regulation. Thus, enhancing self-efficacy requires not only improving the quality of simulation but also creating educational environments that foster major satisfaction.

Third, simulation quality was found to have a strong positive effect on clinical performance, with approximately 50% of the variance explained. This supports previous evidence that simulation is highly effective for integrating knowledge, skills, and attitudes necessary for clinical practice (26, 51). In particular, scenario-based training, the use of standardized patients, and structured debriefing offered students repeated and in-depth opportunities to strengthen their clinical performance.

This study has several limitations that should be considered when interpreting the findings. First, a single-group pre-test/post-test design was employed, which limits the ability to draw causal inferences regarding the effectiveness of the simulation intervention. Although this design is appropriate for exploratory and developmental research, the absence of a control or comparison group restricts robust evaluation of intervention effects and increases the potential influence of confounding variables.

Second, while fourth-year nursing students were an appropriate population given the complexity of the simulation scenario, the justification for the sample size was limited. The power calculation was initially based on statistical approaches (e.g., independent groups *t*-test and repeated-measures ANOVA) that are not fully aligned with a single-group pre–post design. Although the final sample size of 50 participants was sufficient for pre–post analyses using multiple regression, this methodological limitation should be acknowledged.

Third, randomization was not applicable due to the single-group design, and therefore selection bias cannot be entirely ruled out. In addition, the target population was restricted to senior nursing students from a single institution, which may limit the generalizability of the findings to other educational settings or student populations. Furthermore, as the study relied on self-reported measures, the possibility of measurement bias, including response bias and social desirability effects, should be considered.

Future studies should employ more rigorous designs, including appropriate control or comparison groups, clearer population definitions, and power calculations aligned with the chosen analytical approach, to strengthen causal inference and external validity. Furthermore, as the study relied on self-reported measures, the possibility of measurement bias, including response bias and social desirability effects, should be considered. In addition, effect sizes and confidence intervals were not consistently reported, which may limit the interpretation of the magnitude and precision of the findings.

#### 4.2.1 Overall implications

In summary, this study found that home-visit nursing simulation improved nursing students’ self-efficacy and clinical performance, while clinical judgment improved mainly within groups. Simulation quality was identified as a key determinant of clinical judgment and clinical performance, whereas self-efficacy was more strongly influenced by major satisfaction. These results underscore the value of simulation-based education as an effective teaching-learning strategy in community health nursing, particularly in preparing students to care for frail older adults. Future research should focus on developing more complex scenarios and advanced debriefing methods, while also cultivating educational environments that enhance students’ major satisfaction and learning motivation to further strengthen both clinical judgment and self-efficacy.

## 5 Conclusion

This study examined the effects of a home-visit nursing simulation program for frailty prevention on nursing students’ competencies. The findings indicated that clinical judgment and clinical performance showed significant improvements within the intervention group, with perceived simulation quality being positively associated with both competencies. Although self-efficacy also showed an improvement over time, it appeared to be more strongly associated with major satisfaction than with simulation quality.

These results confirm that simulation-based education is an effective strategy for enhancing practical competencies in community health and home-visit nursing. Importantly, simulations focusing on frail older adults address a gap in the literature and provide meaningful empirical evidence for nursing education. Future studies should develop more complex scenarios, implement advanced debriefing strategies, and foster educational environments that enhance students’ major satisfaction and learning motivation, thereby further strengthening clinical judgment and self-efficacy.

## Data Availability

The raw data supporting the conclusions of this article will be made available by the authors, without undue reservation.
